# The role of Lesotho community pharmacists in preventing and controlling cardiovascular diseases: The perceived facilitators and barriers

**DOI:** 10.1371/journal.pone.0301525

**Published:** 2024-04-04

**Authors:** Nthabiseng Florina Motlohi, Kofi Boamah Mensah, Neelaveni Padayachee, Ruwayda Petrus, Varsha Bangalee

**Affiliations:** 1 Discipline of Pharmaceutical Sciences, University of KwaZulu-Natal, Durban, South Africa; 2 Faculty of Pharmacy and Pharmaceutical Sciences, Kwame Nkrumah University of Science and Technology, Kumasi, Ghana; 3 Department of Pharmacy and Pharmacology, University of Witwatersrand, Johannesburg, South Africa; 4 Discipline of Psychology, University of KwaZulu-Natal, Durban, South Africa; Flinders University, AUSTRALIA

## Abstract

**Background:**

Cardiovascular diseases are a leading cause of mortality globally. The impact of cardiovascular diseases can be minimized by addressing modifiable risk factors at primary health care level. Community pharmacists are well-positioned to identify patients at risk of cardiovascular diseases for early detection and initiation of treatment. However, the role of Lesotho community pharmacists in preventing and controlling cardiovascular diseases is not well understood. The purpose of this study was to explore the Lesotho community pharmacists’ role in preventing and controlling cardiovascular diseases.

**Methods:**

The methodological reporting of this study was guided by the consolidated criteria for reporting qualitative studies. A list of registered community pharmacists was obtained from the Ministry of Health. Pharmacists were selected based on their close proximity to the researcher and invited to participate. Semi-structured interviews were conducted until data saturation was reached. The interviews were audio-recorded, transcribed verbatim, and analysed thematically.

**Results:**

Five themes were identified namely: 1) Current roles 2) Future role 3) Facilitators, 4) Barriers, and 5) Community pharmacists’ perceptions of their roles. Generally, community pharmacists were involved in medication counselling, health promotion, and referral of patients. Lack of support from government, patients’ lack of adherence, poor interprofessional relationship, and lack of clear community pharmacy practice guidelines were identified as barriers. Despite the challenges, community pharmacists are motivated by patients’ gratitude for their services.

**Conclusions:**

Lesotho community pharmacists can potentially improve cardiovascular diseases’ health outcomes at primary healthcare level through early detection of CVD risk factors, and health promotion.

## Introduction

Cardiovascular diseases (CVDs) are a group of non-communicable diseases of the heart and blood vessels [1]. CVDs are a leading cause of mortality globally. Approximately 31% of global mortality was attributed to CVDs in 2012, and 33% affected population was below 70 years of age, thus imposing suffering and economic difficulties, particularly in low- and middle-income countries, which carry the highest CVD burden [2]. Over the past two decades, a substantial decline in mortality was realised in High-Income Countries (HICs) through improved prevention and treatment strategies. Contrarily, low- and middle-income countries suffer the most CVD burden with rates twice as much as the HICs in recent years [2]. The World Health Organisation has warned of a projected global mortality increase of 27% by 2030, suggesting immense strain on health systems. Lesotho is a Sub-Saharan African country and is classified under low- and middle-income countries [3]. The country is faced with a growing burden of CVDs which are leading cause of mortality among non-communicable diseases. Between 1990 and 2015, Lesotho experienced over 60% increase in CVD burden [4]. In 2016, the country reported 32% of total mortality due to non-communicable diseases, with CVDs contributing 44% of total non-communicable disease-related deaths [5]. Moreover, in 2019, 45% overall non-communicable disease-related mortality was reported, which represented a 13% increase in three years (2016–2019) [6]. The increase in disease occurrences put a significant strain on healthcare systems and a subsequent poor health outcomes [7]. It is thus imperative that effective strategies to prevention and control of CVDs should be a priority in Lesotho.

Management of CVDs includes detection, screening, treatment, and providing palliative care for the people who need it [8]. Effective strategies to reduce the burden of CVDs can be classified into population-wide interventions to reduce overall risk factor exposure, individual approaches to modify risk factors for high-risk individuals, and treatment of CVD events with the last two being applicable for the primary healthcare setting [2]. At the primary healthcare, a further three stages have been proposed to manage CVDs namely: primary prevention, secondary prevention and early detection [2]. Cardiovascular diseases are highly preventable. The impact of CVDs can be minimized by addressing identifiable and modifiable risk factors such as the use of tobacco, addressing unhealthy diets and obesity, physical inactivity, and the harmful use of alcohol. The outcomes of these risk factors may present in patients as noticeable signs (e.g., high blood glucose, high blood lipids, high blood pressure, overweight and obesity) that can be measured and identified early at primary healthcare facilities. While various healthcare providers have a crucial role to play in mitigating the burden of CVDs at primary healthcare, community pharmacists are easily accessible health professionals and are well positioned to identify "at-risk" patients through medication regimes given their locality.

Community pharmacies are an essential part of primary healthcare. They are located at the heart of communities, and are the first point of contact for many [9]. Community pharmacists can provide both effective population-based and individualized primary healthcare services. The effectiveness of community pharmacist’ roles in primary healthcare interventions is demonstrated in smoking cessation programs, health promotion, and medication therapy management and adherence services [10–15]. Thus, community pharmacy presents an opportunity in primary healthcare setting for effective prevention and control of CVDs. The practice of community pharmacy was rare in Lesotho until late 2000 after the first batch of Bachelor of Pharmacy (BPharm) students graduated from the National University of Lesotho, the only University that produces pharmacists in the country [16]. To date, the country produces around 40 graduates annually, thus suggesting pharmacists as one of the abundant and sustainable health professions in Lesotho [17].

In response to the global agenda to reduce CVD-related mortalities, Lesotho has developed the National Health Policy and the National Health Strategic Plan [18]. The National Health Policy takes into consideration the United Nations sustainable development goals and aims to achieve sustainable development goal 3, and delivery of quality healthcare services to achieve universal health coverage objectives by 2030. To strengthen the country’s response to the prevention and control of non-communicable diseases such as CVDs, Lesotho has proposed the mobilization of human, financial and technical resources through a multi-sectoral inclusive primary healthcare model [7]. The proposed structure presents an opportunity to expand the roles of untapped healthcare resources such as community pharmacists to increase primary healthcare services to achieve better healthcare for all. The government of Lesotho recognizes community pharmacies as an important part of the private-for-profit healthcare system that provides care and medicines [19]. However, there is a paucity of local research on community pharmacists’ role in preventing and controlling non-communicable diseases, particularly CVDs. An understanding of Lesotho community pharmacist’s role in preventing and controlling CVDs is important to policymakers, civil societies, and patients to enable informed decision making in developing effective strategies to prevent and control CVDs. The current study is part of a large mixed method study that seeks to develop a conceptual framework for the integration of community pharmacist in primary healthcare model in Lesotho. The overarching aim of the study was to explore the Lesotho community pharmacist’s role in preventing and controlling CVDs. Specifically, the study seeks to answer the following research questions:

1. What are the roles of community pharmacists in the prevention and control of CVDs in Lesotho?
2. What are Lesotho community pharmacists’ perceived facilitators and barriers towards their contribution in preventing and controlling CVDs?

For the current study, the preventive roles were community pharmacist’s services for patients with reported CVD risk factors such as hypertension, diabetes, and dyslipidaemia but without established CVDs. The control roles (contributions) were community pharmacist’s services for patients with reported established CVDs, while CVD patients are patients with both CVD risk factors and established CVDs.

## Methods

### Study design

The methodological reporting of this study was guided by the consolidated criteria for reporting qualitative studies [20]. The study utilised an exploratory descriptive qualitative research design approach to satisfy the study objectives. In-depth one-on-one semi-structured interviews were employed to establish community pharmacists’ perceptions on their role in preventing and controlling CVDs in Lesotho. Qualitative research is based on constructivism philosophical underpinnings. The concept of constructivism seeks to obtain an in-depth understanding of a phenomenon through individuals’ shared understanding of and experiences in a natural setting, and attempts to create meaning in patterns from the generated data (phenomenology approach) [21]. Little is known about the role of community pharmacists in preventing and controlling CVDs in Lesotho. The researchers found a qualitative study approach a suitable method to explore this phenomenon and to develop a thorough, detailed account of community pharmacists’ views and perceptions of their role in preventing and controlling CVDs in Lesotho.

### Study setting, participants’ recruitment, and sampling

The study was conducted in Maseru and Berea districts in Lesotho. Lesotho is a low- and middle-income country in Sub-Saharan Africa with a population of approximately 2 million people [3,22]. Geographically, Lesotho comprises mostly plateaus, hills, and mountains (highlands), and the lowlands where Maseru (capital city) and Berea districts are located. Administratively, the country is divided into 10 districts. The majority of community pharmacies (90%) are established in four districts in the lowlands including Maseru and Berea [7]. A database of community pharmacies with the name of the responsible pharmacist and contact details was obtained from the Ministry of Health of Lesotho. Participants were selected based on their close proximity to the researcher, their availability, and their willingness to participate. The participants were contacted through phone calls with an email follow up to invite them to participate in the study. Upon acceptance of the invitation, participants were requested to sign an informed consent form as well as facilitate the signing of a gatekeeper’s agreement by the pharmacy owners. A scheduled interview was agreed upon by the interviewer and the participant and the mode of the interviews based on the participant’s convenience.

### Inclusion criteria

The following criteria formed the basis for the inclusion of community pharmacists in the study:

- Had 6 months or above work experience in a community pharmacy.
- A community pharmacist recognised by Lesotho’s Ministry of Health.
- Working full time or part time at a registered community pharmacy in Lesotho.

### Exclusion criteria

- The following formed the basis for the exclusion of participants:
- Community pharmacists who were pharmacy owners but were not directly working in the front shop/with patients.
- Community pharmacists who were not willing to participate.

### Data collection

Data was collected from 15th January 2022 through 27^th^ July 2022. A modified semi-structured interview schedule was adopted from a similar study [23]. Permission to use the schedule was granted by the authors. The interview schedule composed of fifteen open-ended questions and probes to provide cues. The interview started with general questions such as “how long have you been working at a community pharmacy” to establish a rapport with the participants. The core discussion sought to unearth the role of community pharmacists in preventing and controlling CVDs, and facilitators and barriers towards providing services to CVD patients. The interview schedule was pilot tested with two community pharmacists and minor modifications were made on the questions to improve clarity. NFM, the first author, conducted three face-to-face interviews at the participants’ workplace, and eight virtually through WhatsApp calls or Zoom meetings [24,25]. NFM is a pharmacist and a Master of Technology in Pharmaceutical Sciences degree holder, and a current Doctor of Philosophy student. The interviews were recorded using a cell phone and a laptop as a backup through zoom and/or otter.ai applications [25,26]. The interviewer took notes on a printed interview guide during the interviews. The interviews were conducted until data saturation was reached meaning no new themes emerged [27].

### Data analysis

In line with the phenomenology research approach utilized in this study, the researchers found thematic analysis appropriate for the analysis of data. Given that the aim of the study was to explore the community pharmacists’ role in preventing and controlling CVDs, thematic analysis offered a platform to identify opinions by participants and inductively unpack their shared ideas based on their lived experiences and thoughts, and combine the ideas with similar meanings into categories named themes [28]. Thematic analysis is appropriate for most research questions that seek to acquire insights on participants’ experiences and understanding of a phenomena, and compatible with most frameworks and various data collection approaches such as interviews [28,29]. Its flexibility makes it a method of choice for novice qualitative researchers [28]. Data analysis adopted an iterative process by analysing data while collecting it. The researchers transcribed the interviews, coded the data, and identify themes concurrently during data collection. This approach can enhance the researchers’ engagement with the data and facilitates their deeper understanding and relevance of the data collected, thus it allows ongoing adjustments to ensure a more nuanced and understanding of the phenomenon. The interview audio-recordings were transcribed verbatim by the interviewer and the first author (NFM) after a set of two to three interviews. The transcripts were verified for accuracy against the audios by RP and VB. Data analysis followed a framework approach, a 6-step process of “*1) familiarisation*, *2) generating codes*, *3) construction of themes*, *4) reviewing potential themes*, *5) defining and naming themes*, *and 6) producing the report*,” as outlined by Virginia Braun [28]. Data familiarisation started during data collection and transcribing by NFM and continued during data analysis through reading and re-reading of the transcripts, allowing the researcher to immerse in data. Keywords/phrases responsive to the interview questions were highlighted on the first two interview transcripts by NFM, RP and VB. A similar approach was adopted independently by NFM on subsequent interview transcripts followed by discussions and verification with the research team. This facilitated generation of codes (e.g., type of service). The first author (NFM) coded data and RP and VB verified the codes against the transcripts. Coding was done iteratively and inductively through researcher’s reading and re-reading of the interview’s transcripts guided by research questions and identifying meanings in the participants’ responses. Data was then organised around similar meanings and labelled (e.g., blood pressure and cholesterol testing were coded screening tests). Similar codes were organised and re-arranged into themes (e.g., screening tests were labelled current role of community pharmacists (theme 1). The above steps were repeated to all interview transcripts, labelling any emerging theme based on the initial thematic framework or adding and freshly labelling them as new independent themes. Themes were further broken down into sub-themes or re-named to ensure that the themes carry a central meaning around the codes and are supported by the transcripts’ extracts. The final themes and sub-themes were verified and agreed upon by all the authors (NFM, RP, VB, KBM, and NP) to ensure rigor and trustworthiness of the themes and that the identified themes represented the dataset. The coding and generation of themes was done manually. Data were compiled in a Microsoft excel sheet in a tabular form [30]. The first author (NFM) is a doctoral student and a trained pharmacist. The co-authors hold a doctoral qualification in pharmacy and psychology. They have a broad experience conducting qualitative research in pharmacy practice, psychology and other health disciplines.

### Data handling and management

The interview audio-recordings and transcripts were stored in a password-protected computer. Community pharmacists were identified with codes in written transcripts e.g., CPM001. Identity information of the participants including their names, name of community pharmacies and their locations, contact details and an assigned code were saved in a password-protected computer.

### Ethical considerations

The study’s protocol was approved by Humanities and Social Sciences Research Ethics Committee (HSSREC) of the University of Kwazulu-Natal (Ref: HSSREC/00003205/2021) and the Research and Ethics Committee of the Ministry of Health of Lesotho (Ref: ID101-2021). Study information leaflet was emailed to all participants to familiarise themselves with study contents and their rights as participants. The study information and an outline of the participant rights were read out before the interview. All potential participants gave consent before partaking in the study by signing a consent form for face-to-face interviews or giving a verbal consent for online interviews which was audio-recorded. Pharmacy owners granted permission by signing a gatekeeper’s agreement. Participation in the study was voluntary and depended on the willingness of the participants to partake in the study.

## Results

In total seventeen (n = 17) community pharmacists were invited to participate in the study. One pharmacist declined the invitation. Out of sixteen pharmacists who agreed to participate, only eleven were interviewed. The other five were not available for the interview. On average, the interview took 40 minutes, ranging from 27 to 60 minutes. Five females and six males had one to thirteen years of community pharmacy work experience. A total of nine (n = 9) respondents were working in community pharmacies in Maseru urban, one in Maseru rural and one from outside Maseru.

A total of five themes with their corresponding subthemes (for some) were identified from the transcripts after reiterative review of the identified codes, and they are 1) Current roles of community pharmacists, 2) Future role of community pharmacists, 3) Facilitators, 4) Barriers, and 5) Community pharmacists’ perceptions of their roles (Table 1).

**Table 1:** Themes, sub-themes and generated codes in role of community pharmacists in preventing and controlling CVDs.

Themes	Sub-themes	Codes
1. Current roles of community pharmacists		Medication dispensing and counselling Screening tests Lifestyle counselling Patient referral Disease education Patient self-management Medication therapy management Patient follow-ups Keeping patient record databases
2. Future role of community pharmacists	Current role improvement Role expansion	Screening tests Lifestyle counselling Disease education Patient self-management Community outreach services Prescribing
3. Facilitators/enablers		Training Passion (attitude) Patient feedback Convenience Flexible time
4. Barriers	Internal factors External factors	Limited staff Lack of screening equipment Lack of regulations and clear practice guidelines Lack of government support Unclear Medicine supply and access regulations Patient attitude Poor inter-professional relationships
5. Community pharmacists’ perceptions of their roles		Challenging Interesting Confident

CVDs = Cardiovascular diseases.

### Current roles of community pharmacists

A detailed account of the respondents’ quotes supporting the identified themes are represented in Table 2. The study findings showed that community pharmacists in Lesotho provide services daily to both patients with CVD risk factors and established CVDs, highlighting their potential contribution in primary and secondary prevention of CVDs (Table 2, 2.01, 2.03). The identified current roles were predominantly screening tests such as blood pressure tests, medication dispensing and counselling, lifestyle counselling such as diet modification, and referral of patients (Table 2, 2.01). Although less common, respondents mentioned that they provide other services such as patient follow up, keeping patient medical records, patient self-care advice, over the counter dispensing of supplements, CVD education, and medicine therapy management (Table 2, 2.02–2.06).

**Table 2:** Community pharmacists’ interview excerpts.

Excerpt no.	Excerpts
2.01	*“I think almost every day*, *I meet more than 20 patients needing help with cardiovascular problems especially blood pressure and sometimes heart failure…I get prescriptions…I serve them*. *I counsel patients…sometimes I take blood sugar level and blood pressure and check and then counsel and refer patients*.*” CPM008*.
2.02	*“…a preventative approach in the way of education about the risks that may come with certain lifestyles and how they may expose them to cardiovascular for instance*, *weight management*, *smoking*, *the type of food that they eat*, *exercises and so on*. . . .*" CPM003*.
2.03	*“The most common advice that I give to patients is the issue of diet and exercise…and also advice on the supplements maybe that could help them that I think that is what I normally do*.*” CPM007*.
2.04	*“…people will be taking the medication and they will be experiencing side effects*, *and as a pharmacist you feel like no*, *this medication has to be changed*. *We communicate with the doctor who prescribed the medication*. *We discuss with them on helping the client so that the medication can be changed” CPM011*.
2.05	*“…we sell glucometers for them to monitor their sugar levels at home*. *We advise people who can afford…to have a small booklet that…can actually record their readings*, *maybe they can take their reading during the morning and in the evening*.*” CPM001*.
2.06	*“I play a role in advising them on proper compliance to medication*, *the importance of them going back for check-up so that the doctor can really assess whether the medication is still okay or not*.*” CPM007*
2.07	*“…diagnostic tests can be improved in the future … [currently] I am able to test for the blood pressure only*. *So… [other tests like] cholesterol and the others…those would assist…in terms of prevention*, *promotion…we are giving medication to patients…so in future if we can provide services that will promote good health*, *making pamphlets…” CPM007*.
2.08	*“…pharmacists can be engaged in providing educational materials…either within or outside the pharmacy…pharmacists can engage their patients on nutrition…we need to intervene on how to proper manage their weight and also their lifestyle…” CPM002*.
2.09	*“I think the pharmacists could go to the radios and media outlets*, *teach everyone about them and about cardiovascular diseases*, *their prevention and their long-term complications*.*” CPM008*.
2.10	*“…there should be some costs introduced or the prescribing costs so that community pharmacists can be able to diagnose and prescribe the medication for the patients*.*” CPM010*.
2.11	*“We are confined in one space and setting…maybe it’s high time we mobilise our services so that we at least reach almost everyone*.*” CPM004*.
2.12	*“We are trained to offer counselling to help people with cardiovascular diseases and to help manage it…everything that we were taught and advised on at school and at the site of practice is enough*.*” CPM008*.
2.13	*“A qualification alone is not enough*. *We can be qualified and be filled with a lot of theory*. *But now*, *when we talk about a community pharmacist*, *we talk about a practical part of pharmacy*. *So*, *you can’t say I am now a qualified community pharmacist*, *and I don’t need personal improvements*.*” CPM011*.
2.14	*“You continue doing good…I think it’s about the passion and understanding your importance to the community as a community pharmacist*.*” CPM011*.
2.15	*“It’s interesting because you get immediate fulfilment*. *Every time you help somebody*, *and they come back*, *and they thank you because they got better*. *It’s quiet fulfilling*.*” CPM009*.
2.16	*“The feedback that you get*, *after you have assisted the patient is the one that is motivating to continue to educate them*.*” CPM007*.
2.17	*“So*, *you see that I get a lot more people actually coming to services because they know it’s close-by…we have a very small consulting room where we do our tests…blood pressure…sugar*, *diabetes*, *all the consulting work…I do it in there” CPM001*.
2.18	*“I do not see so many patients*. *I have time to counsel patients*. *We have a clinic where we take blood sugar and blood pressure…” CPM008*.
2.19	*“…in some places*, *only a community pharmacy exists…I think pharmacists are well placed to be able to address the needs…especially in remote locations*.* *.* *.*with a community pharmacy at every corner…there isn’t a lack of access…with regards to cardiovascular disease*.*” CPM003*.
2.20	*“I think actually there is spacious time somehow*. *It is able to accommodate patients those that are going to and coming from work*. *We are able to accommodate everybody within those particular opening hours*.*” CPM002*.
2.21	*“We open at 7 am and knock off at 7 pm*.*” CPM011*.
2.22	*“There is some congestion of patients coming in*. *So*, *while advising the patient on CVD events*, *you are also looking at the time*. *The time is limited*. *From my experience*, *its shortage of staff and so we can’t easily pay attention to patients…” CPM002*.
2.23	*“At the moment we don’t have the machinery to check whether they are high blood pressure… currently our BP machine is not functioning*.*” CPM010*.
2.24	*“…we don’t really have good government intervention of such because there are certain subsidies that I think if we are given such…then it’s easier…to engage in certain things that you would want to do with patients…they’re gonna say it’s expensive to do that*. *So it would be nice when government subsidises certain things such as employees’ salaries*.*” CPM001*.
2.25	*“Not being able to access some of the medication*. *You find that suppliers from [neighbouring country] have decided to regulate or control some of the items*. *Medications that we were getting easily*, *it has become difficult to get or it’s not possible to get*.*” CPM009*.
2.26	*“The first thing that is challenging…there are no laws that govern the practice of pharmacy in retail*. *So*, *that says that normally individuals work based on the ethics that they feel are appropriate*.*” CPM007*.
2.27	*“…because as much as sometimes I can say there’s a there’s always going to be a conflict…because our rules and laws are not very clear and nobody’s abiding by those*.*” CPM001*.
2.28	*“…the impatience of the patient*. *They wouldn’t even give you time to listen*, *they would tell you they are in hurry…they are not interested*.*” CPM008*.
2.29	*“Some of them*, *even if they have got blood pressure*, *they don’t take their medication… especially men*, *they do not comply*.*” CPM005*.
2.30	*“…for those who are less fortunate has been access to good food*, *because they eat what they can*. *So now when a patient comes in who is earning so little and you tell them to change their lifestyle…to buy something that is healthier…it means it’s more expensive because now they must eat vegetables*.* *.* *.*and that is difficult*.*” CPM001*.
2.31	*“The cost of the medication can also affect the clients taking the medication*. *Other people come to me and said they could not take the medication for three days because they did not have money to buy them*.*” CPM011*.
2.32	*“We are not talking*. *We just refer the patients without talking to the people who will be helping them there*. *Sometimes we don’t know what happens to the patients” CPM010*.
2.33	*“… we are able to make recommendations to the patients to see the doctor*. *We are also able to scrutinise the patients’ prescriptions and then advise the relevant doctor with the possible medication interaction…but the feedback I don’t think it suits both of us… you would find that patients are still not being changed on some medications they are still experiencing some problems with their medication*, *and communication is basically not that good*.*” CPM002*.
2.34	*“…it is very interesting and challenging because it requires a health professional to be familiar with some guidelines…you are a hero*, *the patients that are visiting you trust your services*. *You are loved by many people*. *You meet the needs of many people that you serve*. *It’s a very good feeling…” CPM011*.
2.35	*“I feel confident to give advice to patients to improve their lives*.*” CPM010*.
2.36	*“…for me I am confident about such conversations because I understand that the lifestyle modification plays a bigger role in avoiding lots of CV events*.*” CPM002*.
2.37	*“… most of the advice we give at pharmacy setting is free*. *So*, *if we start providing such services*, *it should be at a fee also to encourage professionals who are providing the services*, *so that they don’t feel like their time is wasted*. *They know that this is the service they are going to charge*. *They will be more dedicated*.*” CPM009*

CV = Cardiovascular, CVDs = Cardiovascular diseases.

### Future role of community pharmacists

Respondents perceived that they are not exercising their full potential as community pharmacists as they believed their role can be expanded to include more services in preventing and controlling CVDs. Lesotho Community pharmacists believed that although they are providing services to CVD patients (Table 1), their services are limited, and they have an opportunity to provide a broad range of screening tests. For instance, some respondents provided blood pressure testing only and they saw an opportunity to providing other screening tests such as cholesterol (Table 2, 2.07). While some respondents indicated that they offer routine weight management to CVD patients, it presented an opportunity for future expansion of services for other pharmacists (Table 2, 2.08). Additionally, the pharmacists stated that they should increase coverage of their CVD education by using various platforms such as mass media (Table 2, 2.09). The pharmacists highlighted that they provide some the health promotion services at no extra charge to the patient and believed that charging such services will motivate them (Table 2, 2.37). Besides enhancing services that they are already providing, respondents believed that their provisions should be expanded to include prescribing and community outreach services at a fee (Table 2, 2.10–2.11).

### Facilitators

The respondents believed that their ability to provide services in preventing and controlling CVDs is facilitated by their professional training. They believe that their educational training has equipped them with skills to provide services to CVD patients (Table 2, 2.12). Contrarily, some respondents indicated that formal training should be complimented with continuous professional training (Table 2, 2.13). On the other hand, some community pharmacists believed that their passion to serve the public is the main driver (Table 2, 2.14). Other respondents perceived a warm relationship with and positive feedback from their clients make it easier for them to provide services to CVD patients (Table 2, 2.15–2.16).

Furthermore, pharmacists perceived community pharmacy services as convenient due to their location in the community which makes them to be easily accessible (Table 2, 2.19). The presence of a consulting rooms provides private space for screening tests and counselling (Table 2, 2.18). Correspondingly, they stated that their opening hours (7 am– 7 pm) offer flexibility to the patients and accommodate CVD patients to visit a community pharmacy beyond normal working hours (8 am– 5 pm), while others indicated that they have enough time to spend on counselling patients (Table 2, 2.20–2.21).

### Barriers

Lesotho community pharmacist’s provision of services in preventing and controlling CVDs comes with challenges. The identified barriers were categorised into two subthemes namely: internal and external barriers.

#### Internal barriers

For the purposes of this study, internal barriers are those that the pharmacist has control over and can change. Some respondents stated that they encounter many patients, and they are not able to provide satisfactory services to CVDs due to limited staff (Table 2, 2.22). Similarly, lack of screening equipment such as sphygmomanometer hinders their provision of services to CVD patients (Table 2, 2.23).

#### External barriers

For the purposes of this study, external barriers are influenced by external factors that the community pharmacy does not have direct control to change. The respondents stated that they do not get sufficient support from the government. Additionally, unclear medicine supply and access regulations encourage regular medicine stockouts (Table 2, 2.24, 2.25). Similarly, the absence of legislation that controls and enforces community pharmacy’s scope of practice encourages work conflict between community pharmacists and other healthcare professionals (Table 2, 2.26–2.27). Other community pharmacists believed that CVD patients’ attitude and behaviour such as non-adherence and lack of time, add to the challenges they encounter (2.28–2.29). On the other hand, patients are willing to cooperate and consider the community pharmacist’ advice but fail to comply because of financial constraints (Table 2, 2.30–2.31).

Regarding community relationship with other healthcare professionals, it was evident that there were some community pharmacists who were enjoying good relationship with medical doctors and nurses involved in CVD care, while others perceived inter-professional relationship as poor with lack of communication (Table 2, 2.32–2.33).

### Community pharmacists’ perceptions of their roles

Community pharmacists had a positive attitude about their role in health promotion services in the management of CVDs. They perceived their role as challenging but interesting and regarded the experience as a learning platform (Table 2, 2.34).

Moreover, community pharmacists regarded themselves as confident in delivering health promotion services such as weight management counselling (Table 2, 2.35–2.36).

## Discussion

To the best of the authors’ knowledge, this is the first study to report the role of community pharmacists in preventing and controlling CVDs in Lesotho. Lesotho is not an exception to the global burden of CVDs particularly in low- and middle-income countries. The country has seen a considerable increase in CVDs in the last two decades [4,6]. Thus, it is imperative that prevention and control of CVDs should be a priority in Lesotho’s health systems. With the rising morbidity rates, and shortage of skilled human resources in healthcare, community pharmacists stand an opportunity to become part of the proposed multi-sectoral model towards prevention of CVDs.

The study findings established that the pharmacists provide services to both patients with CVD risk factors (e.g., hypertension) and patients with established CVDs (e.g., heart failure). This suggests that contribute in primary and secondary prevention of CVDs, and detection of modifiable risk factors. Generally, the role of community pharmacists in CVD prevention and control involves the traditional medication dispensing, detection, and health promotion. Previous reviews have reported important clinical improvement of CVD risk factors following community pharmacist interventions. In a systematic review conducted by Chiazor, Evans [13], community pharmacist’s interventions on diabetes and hypertension revealed favourable clinical outcomes [13]. Similar findings reported substantial improvement on hypertension, diabetes, hyperlipidaemias, obesity, and smoking cessation following community pharmacists’ interventions [10–12,14,15]. The identified roles of community pharmacist suggest that they can provide expanded services and stand an opportunity to improve CVD burden in Lesotho. The effectiveness of the services opens room for future studies.

In health promotion, the most mentioned service was weight management counselling. However, patient adherence to weight loss programs were discouraged by affordability where patients could not afford the recommended eating habits against their willingness to change their lifestyles. In some instances, patients were not willing to receive counselling, but would prefer to get their medication and leave. This suggests that the lifestyle modification counselling services by a community pharmacist could only reach a few patients who were willing to receive or could afford such services. The reasons behind pharmacist perceived patients’ hesitation and lack of willingness to receive needs further investigation. On the same note, it was evident that the community pharmacist would initiate the lifestyle discussion on many occasions, posing the question of patients’ awareness of community pharmacist’s provision of service beyond medication dispensing. A similar finding was reported by Peletidi, Nabhani-Gebara [31] who established that Greek pharmacists adopted a paternalistic approach where pharmacists give instructions to the patients [31]. On the other hand, patients initiate lifestyle conversations in the United Kingdom (UK). There is a need to investigate patients’ views about the role of community pharmacists in preventing and controlling CVDs in Lesotho to better understand patients’ views of community pharmacy services. Nonetheless, one of the drivers of community pharmacist’s continued provision of services towards CVD patients was a good relationship with their patients and trust developed by patients towards pharmacists. There is evidence that the positive feedback the pharmacists receive from the patients was an important enabler. The variability in patients’ attitude towards community pharmacists’ services calls for future studies to establish patient’ perceptions of community pharmacist’s services in Lesotho. The quality of services provided by community pharmacists is evaluated based on the economic (cost implications), clinical (events that occur following disease occurrence or therapy), and humanistic (patient-centred outcomes including patient satisfaction, quality of life) outcomes (ECHO) model [32]. According to Theory of Planned Behaviour, patients’ behavioural beliefs and attitude hint their intention to utilise healthcare services, contributing towards positive or negative outcomes [33]. Thus, it is imperative to investigate patients’ perception of community pharmacists’ services in Lesotho to encourage patient-centred outcomes.

While it was evident that dispensing of medication was a standard practice, other services such as screening were not provided by some of the facilities partly due to lack of screening equipment. Interestingly, most community pharmacists provided blood pressure screening. Only few respondents indicated that they provide other screening tests such as cholesterol. While limited provision of screening services could possibly be a result of lack of equipment, the absence of clear regulations and guidelines cannot be overlooked as a contributing factor for the lack of standardisation in provision of services at the community pharmacies. Nonetheless, the findings compare with practice in parts of Europe and Australia where counselling on lifestyle modification were the common health promotion activity [31,34].

Community pharmacists revealed that they work under challenging circumstances that hinder their practice scope. Firstly, the study established that patients do not return to the pharmacy after referral to the physicians. This possibly affect continuity of services and possibly the effectiveness of the services provided by community pharmacists due to patients’ loss to follow up. Additionally, this could suggest possible role conflicts and competition between community pharmacists and physicians. Secondly, the inter-professional relationship between community pharmacists and other healthcare professionals is not well understood. While some community pharmacists reported a good relationship with medical doctors, other community pharmacists expressed their frustration over poor communication with doctors due to lack of structured collaboration guidelines. According to World Health Organization [35], every person should receive quality healthcare services from anywhere [35]. The relationship between healthcare professionals involved in CVD care should be regulated to afford patients freedom to obtain healthcare services from anywhere they wish, without being forced to go to a certain pharmacy or clinic. Another barrier was the lack of support from the government. Community pharmacists believed that they could dedicate more time to delivering services to CVD patients should their services get a subvention from the government to pay off staff salaries and medication. Government support could potentially encourage more affordable services and broader utilisation by the public. There is need for standardisation of community pharmacy practice to support universal health coverage objectives. Generally, the barriers stem from legislation that does not provide clear community pharmacy guidelines of service.

Despite the challenges, community pharmacists believed that they have opportunity not only to improve their current services but to expand their services to include for instance, prescription which is normally reserved for medical profession. It is unclear what prompted this desire, which could see role conflicts between pharmacists and other healthcare professionals. Also, this view was echoed by respondents who indicated limited time and staff as barrier of service provision to CVD patients. Expanded services might worsen their current workload and promotes inefficiency. There is need to establish a clear structure of professions and their roles in the country that work collaboratively for improved health outcomes. Moreover, community pharmacists regarded themselves as confident in delivering health promotion activities. This contrasts with views of pharmacists in UK and Greece who expressed their lack of confidence in initiating lifestyle conversations with patients [31]. The perceived confidence might be encouraged by lack of reference point due to lack of guidelines. This further substantiate the need for standardisation of community pharmacy services.

The study had limitations. The results presented might not cover a full view of community pharmacists in Lesotho since all the community pharmacists, but one, were based in the capital city. Those based outside Maseru might share different opinions. Therefore, further research to include a larger representative sample that takes account of demographic factors. Nonetheless, the study provides in-depth views of community pharmacists about their role in preventing and controlling CVDs in Lesotho and adds to the current literature. The participants had a good gender representation of 5:6 females to males and a broad coverage of experience ranging from one to thirteen years. The findings built a foundation for future research and provides insights to healthcare policy makers regarding the role of community pharmacists in preventing and controlling CVDs in Lesotho. The authors are conducting a survey to a generalizable sample that includes other districts beyond the capital city (Maseru).

## Conclusions

Lesotho community pharmacists can potentially improve CVD health outcomes at primary healthcare level through early detection of CVD risk factors, and health promotion services. They stand an opportunity to become part of primary healthcare structure in the management of CVDs. However, there is need for clear guidelines for standardised community pharmacy services and demarcation of the roles for healthcare professionals involved in CVD management. The study established that contact details obtained from the Ministry of Health of Lesotho had unreachable numbers and/or email databases. Further to that, the authors believe that there are more community pharmacies in Lesotho than found in the database since there are pharmacies on every corner as observed by the first author (NFM). It is possible that some pharmacies are not available in the database. Thus, the authors recommend regular update of community pharmacies’ database.
